# Prostate cancer detection after atypical small acinar proliferation (ASAP): A 10‐year single‐centre cohort

**DOI:** 10.1002/bco2.407

**Published:** 2024-07-10

**Authors:** Thineskrishna Anbarasan, Mutie Raslan, Kanchan Ghosh, Philip Macklin, Claudia Mercader, Tom Leslie, Freddie C. Hamdy, Richard Colling, Lisa Browning, Ian Roberts, Clare Verrill, Richard J. Bryant, Francisco Lopez, Alastair D. Lamb

**Affiliations:** ^1^ Oxford University Hospitals NHS Foundation Trust Oxford UK; ^2^ Nuffield Department of Surgical Sciences University of Oxford Oxford UK

**Keywords:** ASAP, atypical small acinar proliferation, biopsy, prostate cancer

## 1 INTRODUCTION

Atypical small acinar proliferation (ASAP), found in 5% of prostate biopsies, represents a focus of atypical cells that fall short of a cancer diagnosis.^1^ ASAP may be associated with a diagnosis of prostate cancer (PCa) upon repeat biopsy in 25%–50% of patients within 5 years.^1^ The proportion of these cases that may be classified as being intermediate‐ or high‐grade PCa varies in the literature, ranging from 6.0% to 22.5%.^2^, ^3^ Until recently, diagnosis of ASAP was an indication for early repeat biopsy in international guidelines. However, recent studies referenced by the European Association of Urology (EAU) guidelines suggest low rates of subsequent Gleason grade group (GG) ≥ 2 PCa, similar to following a previous negative biopsy, leading to a softening of the recommendation for ASAP as an indication for performing early repeat biopsy.^4^ We therefore aimed to test the hypothesis that prostate cancer diagnosed on early re‐biopsy after detection ASAP is always low grade by interrogating a large prospective pathology database. We also aimed to determine the time interval between detection of ASAP and diagnosis of csPCa, if present.

We scrutinised pathology records according to a prospectively derived protocol (ID: CU96T) for all consecutive patients with ASAP on needle biopsy, transurethral resection of the prostate (TURP) chippings, or holmium laser enucleation of the prostate (HoLEP) specimens between January 2010 and November 2021 at a single tertiary institution. We classified pathological upgrading to csPCa as any Gleason pattern 4 disease identified within 2 years of the initial biopsy/TURP/HoLEP specimen detecting ASAP. Where available, we reviewed pre‐biopsy multiparametric MRI (mpMRI) reports for PI‐RADS scores at the time of ASAP diagnosis and obtained the prostate volume in order to derive the PSA density (PSAD). A multi‐variable logistic regression model (including age, PSAD and PI‐RADS) was constructed to determine factors associated with the development of csPCa.

Approximately 13 240 prostate samplings were performed (11 240 needle biopsy and 2000 HoLEP/TURP specimens) over the 10‐year period. ASAP was identified in 617 (4.7%) biopsy samplings, involving 523 patients. Of these, 51 (9.7%) patients had a pre‐existing history of PCa and were excluded from further analysis, leaving a sample size of 472 individuals with de novo ASAP (Table 1). The baseline characteristics of the cohort are summarised in Table 1. Two hundred and thirty‐seven (50.2%) patients had a repeat biopsy (Table S1) within a median of 92 days (IQR: 56–283). The median PSA within 3–6 months of ASAP detection was higher amongst patients who underwent repeat biopsy (6.7 vs. 5.08 ng/ml, *p* = 0.001) consistent with clinical judgement advocating repeat biopsy. In the 248 of 472 (52.5%) patients with pre‐biopsy MRI, logistic regression revealed age <65 years (OR: 3.11; 95% CI: 1.74–5.69), PSAD > 0.15 ng/ml^2^ (OR: 2.06; 95% CI: 1.13–3.80), and PI‐RADS ≥ 3 (OR: 1.88; 95% CI: 1.05–3.42) were independently associated with patients undergoing repeat biopsy following detection of ASAP.

**TABLE 1:** Summary of baseline characteristics of cohort and multivariable regression model for development of GG ≥ 2 PCa amongst patients with ASAP. A total of 74 patients who underwent repeat biopsy and have complete data were included for regression analysis.

Baseline characteristics		
Total (*N*)		472
Age (median, Q1, Q3)		66 (61–71)
Pre‐biopsy PSA (ng/ml) (median, Q1, Q3)		7.25 (5.25–10.75)
PSAD/ng/ml^2^ (median, Q1, Q3)		0.14 (0.10–0.22)
PSAD/ng/ml^2^ (%)	>0.15	135 (28.6)
Repeat biopsy (%)		237 (50.2)
PI‐RADS (%)	1/2	117 (24.8)
	3	78 (16.5)
	4/5	53 (11.2)

Development of GG ≥ 2 PCa		OR (95%CI)
Number developed GG ≥ 2 PCa (*n*)		57
Age	≥65	Ref
	<65	0.55 (0.18–1.62)
PSAD/ng/ml^2^	≤0.15	Ref
	>0.15	3.21 (1.12–9.74)
PI‐RADS	1/2	Ref
	3/4/5	2.35 (0.36–15.4)

Abbreviations: PI‐RADS, Prostate Imaging—Reporting and Data System; PSAD, PSA density.

In the 237 patients who underwent repeat biopsies, intermediate‐ or high‐grade PCa (GG ≥ 2) was found in 57 (24.1%) patients (18 high‐grade [GG 4/5] versus 39 intermediate‐grade [GG 2/3]) within 2 years at a median interval of 128 days (IQR: 61–260). Low‐grade PCa (GG1) was detected in 77 (32.5%) patients. GG ≥ 2 PCa was detected on the ipsilateral side of ASAP diagnosis in 46 of 57 (80.8%) patients, which we therefore hypothesise to be related to the original diagnosis of ASAP. Amongst patients who underwent repeat biopsy following detection of ASAP, mpMRI was performed with PI‐RADS data available for 74 patients (31.2%; from December 2014). Of these, 48 (64.8%) patients had PI‐RADS ≥ 3. A PSAD > 0.15 ng/ml^2^ at time of ASAP diagnosis was independently associated (OR: 3.21; 95% CI: 1.12–9.74) with the detection of GG ≥ 2 PCa (Table 1). A subgroup of 16 patients with pre‐biopsy PI‐RADS 4–5 lesions were not found to have csPCa on repeat biopsy within 2 years. Repeat mpMRI (median interval of 526 days from detection of ASAP) was performed in 14 of 16 of these patients, of which five lesions were downgraded to PI‐RADS 3 and a further five to PI‐RADS 1/2. Expressed as a proportion of all men with ASAP, PI‐RADS score 4/5 was associated with the development of GG ≥ 2 PCa (OR: 5.86; 95% CI: 2.01–19.6), as expected for MRI visible lesions.

There were 103 patients without pre‐biopsy PI‐RADS score (predating regular pre‐biopsy MRI), hence excluded from the above regression analysis, ROC curve analysis showed that the AUC for PSAD > 0.15 ng/ml^2^ was 0.734 (95% CI: 0.642–0.827) for the detection of GG ≥ 2 PCa within 2 years (Figure S1). The positive predictive value (PPV) and negative predictive value (NPV) for PSAD threshold of 0.15 ng/ml^2^ were 68.8% and 83.3%. At a higher PSAD threshold of 0.20 ng/ml^2^, the PPV and NPV were 74.0% and 80.0%, respectively (Table S2) .

The rate of detection of ASAP in our cohort is 4.7%, which is consistent with the rate of approximately 5% previously reported in the literature. Younger patients, those with a raised PSAD, and those with positive mpMRI findings (PI‐RADS ≥ 3) were more likely to undergo re‐biopsy. It is also likely that the decision to re‐biopsy was influenced by guideline recommendations, which have varied during this time period. The rate of clinically important PCa, adopting a definition of any Gleason pattern 4 disease within 2 years, was 24.1%. This is at odds with studies suggesting GG≥ 2 PCa is not diagnosed after ASAP^4^ and is consistent with Kim et al. who reported GG ≥ 2 PCa in 19.6% of patients with ASAP, this being similar to reports in other contemporary studies.^2^, ^5^, ^6^ In a meta‐analysis including 16 studies and 1796 patients, those who underwent repeat biopsy within 6 months of ASAP diagnosis, had lower clinically important PCa detection (9%) compared to those who had repeat biopsy after (22.1%).^7^

On analysis of repeat MRIs, 71.4% of patients with PI‐RADS 4–5 lesions who did not have GG ≥ 2 PCa on repeat biopsy had the PI‐RADS score downgraded to ≤3 upon repeat mpMRI. Although we only focused on ASAP, this is consistent with the report by Meng et al. who observed a PI‐RADS score downgrade of 73% from PI‐RADS 4–5 to ≤3 amongst patients with any benign biopsy sub‐type.^8^ This may suggest that microenvironmental changes associated with ASAP could predispose to false positive mpMRI changes.

PSAD > 0.15 ng/ml^2^ at time of ASAP diagnosis was independently associated with the subsequent detection of GG ≥ 2 PCa at repeat biopsy. A reduced rate of false positives was observed with increasing PSAD thresholds. In a report by Warlick et al., PSAD was an independent predictor of detection of GG ≥ 2 PCa at repeat biopsy within 1 year of a diagnosis of ASAP.^9^ We observed an association between the laterality of ASAP diagnosis and subsequent GG ≥ 2 PCa detection, highlighting importance for adequate ipsilateral sampling given tissue heterogeneity.^10^, ^11^

This study has several limitations. Firstly, the number of men who underwent repeat biopsy will have been influenced by clinical decision‐making. Secondly, we only adjusted for a limited number of baseline clinicopathological variables as per our protocol. Next, the lack of a control group with completely negative biopsies precludes comparison to determine whether ASAP is an independent predictor for development of GG ≥ 2 PCa. Finally, this is single‐centre data reflecting one pathology department's criteria for reporting ASAP.

Overall, we must reject our initial hypothesis that prostate cancer diagnosed after ASAP is always low grade. Contrary to this supposition, we have observed an important rate of detection of GG ≥ 2 PCa following a previous diagnosis of ASAP at needle biopsy or following histological analysis of TURP/HoLEP specimens. These findings support that patients diagnosed with ASAP should be followed up for consideration of repeat mpMRI and/or sampling. Further studies may guide development of risk‐stratification tools based on clinicopathologic factors such as PSAD and pre‐biopsy mpMRI PI‐RADS score at the time of initial ASAP diagnosis. This would enable clinicians to better counsel patients and identify those requiring more stringent follow‐up, potentially with re‐biopsy or repeat mpMRI follow‐up.

## AUTHOR CONTRIBUTIONS

*Conceptualisation:* Mutie Raslan, Claudia Mercader, Francisco Lopez and Alastair D. Lamb. *Data curation:* Kanchan Ghosh, Philip Macklin, Richard Colling, Lisa Browning, Ian Roberts. *Formal analysis:* Thineskrishna Anbarasan, Mutie Raslan and Alastair D. Lamb. *Methodology:* Thineskrishna Anbarasan, Mutie Raslan, Richard J. Bryant, Richard Colling, Clare Verrill and Alastaiir D. Lamb. *Supervision:* Richard J. Bryant, Clare Verrill, Freddie C. Hamdy and Alastair D. Lamb. *Writing (original draft):* Thineskrishna Anbarasan, Mutie Raslan and Alastair D. Lamb. *Writing (review and editing):* Thineskrishna Anbarasan, Mutie Raslan, Kanchan Ghosh, Philip Macklin, Claudia Mercader, Tom Leslie, Freddie C. Hamdy, Richard Colling, Lisa Browning, Ian Roberts, Clare Verrill, Richard J. Bryant, Francisco Lopez and Alastair D. Lamb.

## CONFLICT OF INTEREST STATEMENT

Lisa Browning receives funding for a study (ArticulatePro) evaluating Paige Prostate, which is funded by the NHSX Artificial Intelligence in Health and Care Award: Driving system‐wide improvements with real‐world economics evidence and subspecialist‐led adoption guidelines for full workflow implementation of AI with Paige Prostate cancer detection (Paige AI 2020, New York), grading and quantification tool in histopathology departments (AI_AWARD02269). Richard Colling is part funded by UKRI (WCT WVI MAP project and PathLAKE project in‐kind partnership with Philips), mdxhealth (research funding), NHSX (ARTICULATE PRO: evaluating Paige AI), and the Clarendon Fund (University of Oxford). Richard Bryant receives grant funding for TRANSLATE Trial (NIHR‐HTA: NIHR131233), PART Trial Funding (NIHR‐HTA: 17/150/01), Cancer Research Clinician Scientist Fellowship (A22748). Participates in STAMINA Trial Programme Steering Committee. Ian Roberts receives consulting fees from Novartis and Travere Therapeutics and support for attending meetings from American Society of Nephrology, International Academy of Pathology and Asia Pacific Society of Nephrology. Alastair Lamb receives grant funding for TRANSLATE Trial (NIHR‐HTA: NIHR131233). ADL was supported by a Cancer Research UK Clinician Scientist Fellowship award (C57899/A25812).

## Supporting information


**Figure S1.** Schematic summary of the sub‐group of patients with complete clinicopathologic data available for regression analysis.
**Table S1.** Demographics and clinical characteristics of patients with ASAP with respect to whether repeat biopsy was performed.
**Table S2.** Positive (PPV) and negative (NPV) predictive values at various PSA density (PSAD) thresholds for development of GG ≥ 2 PCa within 2 years of ASAP diagnosis in the sub‐group of patients without pre‐biopsy mpMRI data (n = 103).
