# Supplementary material for: Prostate cancer detection after atypical small acinar proliferation (ASAP): A 10‐year single‐centre cohort
Source: BJUI Compass. 2024 Jul 10;5(9):834–6. doi: 10.1002/bco2.407 (PMC11420094; doi:10.1002/bco2.407)
Supplement: Supplementary file 1 — Figure S1. Schematic summary of the sub‐group of patients with complete clinicopathologic data available for regression analysis. Table S1. Demographics and clinical characteristics of patients with ASAP with respect to whether repeat biopsy was performed. Table S2. Positive (PPV) and negative (NPV) predictive values at various PSA density (PSAD) thresholds for development of GG ≥ 2 PCa within 2 years of ASAP diagnosis in the sub‐group of patients without pre‐biopsy mpMRI data (n = 103). [file BCO2-5-834-s001.docx]

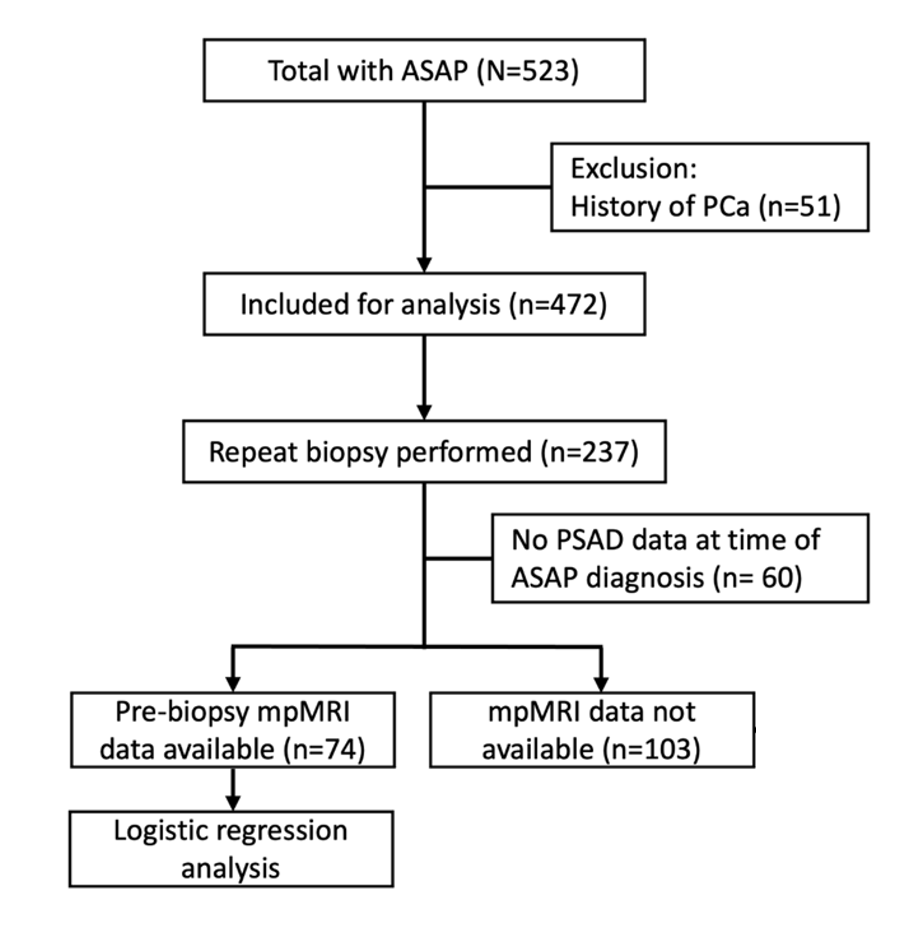


**Supplementary Figure 1:** Schematic summary of the sub-group of patients with complete clinicopathologic data available for regression analysis.

|  | | | |
| --- | --- | --- | --- |
|  |  | **Repeat biopsy** | **No repeat biopsy** |
| **Total (%)** |  | 237 (50.2) | 235 (49.8) |
| **Age (median, SD)** |  | 65 +/- 7.6 | 68 +/- 7.8 |
| **PSAd/ng/ml^2^ (%)** |  |  |  |
|  | >0.15 | 80 (33.8) | 55 (23.4) |
| **PIRADS Score (%)** |  |  |  |
|  | 1/2 | 26 (11.0) | 91 (38.7) |
|  | 3 | 21 (8.9) | 57 (24.3) |
|  | 4/5 | 27 (11.4) | 26 (11.1) |

**Supplementary Table 1**: Demographics and clinical characteristics of patients with ASAP with respect to whether repeat biopsy was performed.

| **PSA Density threshold (ng/ml^2^)** | **PPV (%) (95% CI)** | **NPV (%) (95%CI)** |
| --- | --- | --- |
| **0.10** | 46.8 (40.0-53.7) | 66.7 (43.6-83.8) |
| **0.15** | 68.8 (55.8-79.3) | 83.3 (68.8-91.9) |
| **0.20** | 74.0 (58.7-85.2) | 80.0 (67.4-88.5) |

**Supplementary Table 2**: Positive (PPV) and negative (NPV) predictive values at various PSA density (PSAD) thresholds for development of GG ≥ 2 PCa within 2 years of ASAP diagnosis in the sub-group of patients without pre-biopsy mpMRI data (n = 103).
